# Insights on the Shared Genetic Landscape of Neurodevelopmental and Movement Disorders

**DOI:** 10.1007/s11910-025-01414-w

**Published:** 2025-03-17

**Authors:** Elisabetta Indelicato, Michael Zech, Anna Eberl, Sylvia Boesch

**Affiliations:** 1 Center for Rare Movement Disorders Innsbruck, Department of Neurology, Medical University Innsbruck, Anichstrasse 35, Innsbruck, 6020 Austria; 2 Institute of Neurogenomics, Helmholtz Munich, Neuherberg, Germany; 3 Institute of Human Genetics, School of Medicine, Technical University of Munich, Munich, Germany; 4 Institute for Advanced Study, Technical University of Munich, Garching, Germany

**Keywords:** Neurodevelopmental disorders, Movement disorders, Exome sequencing, Cerebral palsy, Dystonia, *CACNA1A*

## Abstract

**Purpose of Review:**

Large-scale studies using hypothesis-free exome sequencing have revealed the strong heritability of neurodevelopmental disorders (NDDs) and their molecular overlap with later-onset, progressive, movement disorders phenotypes. In this review, we focus on the shared genetic landscape of NDDs and movement disorders.

**Recent Findings:**

Cumulative research has shown that up to 30% of cases labelled as “cerebral palsy” have a monogenic etiology. Causal pathogenic variants are particularly enriched in genes previously associated with adult-onset progressive movement disorders, such as spastic paraplegias, dystonias, and cerebellar ataxias. Biological pathways that have emerged as common culprits are transcriptional regulation, neuritogenesis, and synaptic function.

**Summary:**

Defects in the same genes can cause neurological dysfunction both during early development and later in life. We highlight the implications of the increasing number of NDD gene etiologies for genetic testing in movement disorders. Finally, we discuss gaps and opportunities in the translation of this knowledge to the bedside.

## Introduction

Neurologists, particularly movement disorders specialists, are trained to classify and discriminate disease entities based on the finest details of clinical phenomenology. Beyond the mere exercise of diagnostic skills, the definition of an accurate movement disorder phenotype is the essential starting point for localizing the site of brain damage and guiding appropriate diagnostic workup and symptomatic therapy. With the genetic revolution, the recognition of clear-cut movement disorder syndromes has also been instrumental in the identification of several disease genes in a “forward genetics” approach using linkage analysis. Paradigmatic examples are the discovery of *SGCE* as main gene for the syndrome “myoclonus-dystonia” [1] and *RFC1* as the gene associated with the triad cerebellar ataxia, sensory neuropathy and vestibular areflexia (CANVAS) [2].

In an opposite “reverse genetics” approach, an ever-growing list of monogenic etiologies for early-onset, clinically less well-defined phenotypes, commonly referred to as “neurodevelopmental disorders” (NDDs) has been unveiled [3]. The term NDDs was introduced in the fifth edition of the Diagnostic and Statistical Manual of Mental Disorders (DSM-5) as an overarching category for a group of conditions with onset in the developmental period that result in functional impairment in multiple domains [4]. NDDs include intellectual disability, communication disorders, autism spectrum disorder (ASD), attention deficit hyperactivity disorder (ADHD), specific learning disorder, and motor disorders (including developmental coordination disorder, stereotypic movement disorder, and tic disorders) [4]. The definition of NDDs also extends to other conditions outside the domain of DSM-5, such as cerebral palsy (CP) and epileptic encephalopathies [5]. The frequency of comorbidity among the NDDs is higher than that expected by chance [6] and provides the rationale for lumping them in a clinical continuum [7]. Due to the extremely heterogeneous and mostly non-specific clinical pictures, NDDs are usually tackled with a “genotype-first” approach using chromosomal microarrays and unbiased exome sequencing [3]. The latter tool has greatly accelerated gene discovery in NDDs [8] and highlighted the molecular overlap with other seemingly unrelated phenotypes, such as adult-onset movement disorders [9–15]. As a result, our perspective is gradually changing. From a dichotomous paradigm distinguishing neurodevelopmental dysfunction and neurodegeneration, cumulative evidence outlines a nuanced clinical spectrum due to genetically determined developmental brain dysfunction [6], whose modulating factors remain elusive.

The present review focuses on the shared molecular landscape of movement disorders and NDDs. We begin our discussion with the genetic discoveries in CP, the epitome of disease of the movement and the developing brain. We then review selected biological pathways that emerged as common culprits of neurological dysfunction both in early development and later in life. Finally, we discuss the clinical implications of the increasing NDDs-gene etiologies in movement disorders.

### The Cerebral Palsy Paradigm

Cerebral palsy (CP) is a clinical diagnosis describing neurodevelopmental phenotypes that primarily affect movement and posture [16]. CP is attributed to nonprogressive disturbances occurring early in the fetal or infant brain [16]. Birth asphyxia secondary to intrapartum complications has long been considered its leading cause [17–19]. Large-scale genetic studies using chromosomal microarray analysis and subsequently exome or genome sequencing have challenged this dogma, demonstrating a genetic etiology in 31.1% of cases on average [20]. The diagnostic yield of exome sequencing may approximately double if CP cases without hints of perinatal brain injury according to clinical history and/or brain MRI are selected [21]. When the broad clinical umbrella of CP is re-evaluated based on the clinics, the presence of a hyperkinetic movement disorder phenotype (dystonic and/or dyskinetic) is another predictive factor for a monogenic etiology [22]. Similar to other NDDs [23–26], the rate of *de novo* variants in CP is high [21] and may explain the relatively constant frequency of these disorders associated with reduced fitness despite the improvement of perinatal care in developed countries [27].

Looking at the molecular pathways involved, the most common monogenic etiologies associated with CP cluster in a few complex processes with a key role in neurodevelopment, such as transcriptional regulation (*CTNNB1*,* FOXG1*,* MECP2*), neuritogenesis (*ATL1*,* KIF1A*,* SPAST*,* TUBA1A*,* TUBB4A*), and synaptic transmission (*CACNA1A*,* GNAO1*,* KCNQ2*,* SCN1A*) [20]. Notably, several of these genes have been previously implicated in classic adult-onset movement disorders, such as autosomal dominant *TUBB4A*-related dystonia [28, 29], spastic paraplegia type 4 (*SPAST*) [30], or inherited cerebellar ataxia phenotypes (*CACNA1A*) [31, 32]. In the following sections, we will focus on these three overarching biological processes and their involvement in both NDDs and specific movement disorder phenotypes.

### Transcriptional Dysregulation as Driver of Neurodevelopmental Brain Dysfunction

Complex processes underlying neurodevelopment and neural function throughout life depend on the coordinated expression of myriads of genes in specific cells at the appropriate time [33, 34]. Beyond the large number of players at a purely genetic level, the ultimate phenotypic complexity underlying neural function is determined by a multifaceted regulation of gene expression. Thus, it is not surprising that an increasing number of genes with DNA-, RNA-, and histone-binding functions are emerging in the landscape of NDDs [35].

Sequential expression of different transcription factors in specific time windows drives the differentiation of neural precursors [33]. For example, *NKX2-1* expression in neural progenitors is required for GABAergic interneuron commitment [36] and basal ganglia development [37]. *NKX2-*1 (Mendelian Inheritance in Man (MIM) *600635) is a well-established human disease gene, initially associated with thyroid and lung developmental defects and later, also with neurological symptoms. One group identified five index patients with additional neurological features such as choreoathetosis, muscular hypotonia, ataxia, and developmental delay in the screening of patients with congenital hypothyroidism, which did not respond to substitution with L-thyroxine, prompting a search for a differential diagnosis [38]. In the same year, another independent group published the association of *NKX2-1* variants with the well-known clinical entity “benign hereditary chorea”, a childhood-onset form of chorea not associated with intellectual decline (see also Table 1) [39].

Beyond direct gene activation and repression, chromatin modification offers another level of control on a large scale. DNA-binding proteins that recruit chromatin- and RNA-modifying factors, such as those of the *CHD* family, have an established role in NDDs [23, 40, 41] and an emerging role in movement disorders [11, 42]. Perhaps the most interesting converging biological pathway in NDDs and movement disorders is DNA methylation, a key regulatory process affecting both ends of the life course [34]. In the zygote, a wave of demethylation occurs prior to methylation imprinting [43]. Alterations in this dynamic process have been implicated in NDDs such as Rett, Prader-Willi and Angelman syndromes [34]. On the other hand, the extent of methylation later in life has been shown to be consistent with the concept of an “epigenetic clock” as a strong predictor of life expectancy [44]. Methylation and demethylation of lysine residues on histone tails is a key dynamic chromatin modification that is mediated by specific methyltransferases (KMTs) and demethylases (KDMs) [45]. Twenty-seven *KMT*- and 24 *KDM*-encoding genes are known, and to date, 22 have been associated with NDDs [35]. *KMT2B* (MIM *606834) encodes histone lysine N-methyltransferase 2B, an epigenetic writer that, like other *KMT2* enzymes, modulates transcriptional regulation by methylating a specific lysine residue (K4) of the histone 3 (H3) protein [46]. H3K4 methylation by *KMT2B* is associated with active transcription and plays an essential role in the normal development and maturation of brain circuits involved in motor control [46–48]. The first association between *KMT2B* and human disease was described in patients with childhood-onset isolated dystonia carrying heterozygous loss-of-function variants [49, 50]. Cumulative reports gradually revealed a much broader phenotype in which developmental features may represent the first or predominant manifestation (see also Table 1) [15, 45], as opposed to adult-onset incompletely penetrant dystonia at the other end of the clinical spectrum [51]. Recently, a unique DNA methylation pattern at CpG sites in peripheral blood from *KMT2B* patients was described, as the so-called epi-signature [52]. This unique biomarker corroborated some of the genotype-phenotype correlations observed in *KMT2B*-related disease. For instance, the *KMT2B* missense variant p.Ala1541Val associated with adult-onset dystonia [51] caused more subtle methylation changes compared to truncating variants seen in early-onset, developmental cases [52]. The importance of proper H3K4 methylation dosage in normal development is further highlighted by the involvement of at least six *KMT2* genes in human disease, despite their seemingly redundant enzymatic function [45, 53, 54]. Elucidating the relationship between dysregulated *KMT2* function and neurological disease is of particular interest for the development of therapeutic strategies. Indeed, methylation is a dynamic and potentially reversible or inducible process, as suggested by the striking therapeutic effect of deep brain stimulation in the setting of certain *KMT2B* variants [15].

### Defective Neuritogenesis in Developmental Motor Disorders

Neuritogenesis is a crucial step in neurodevelopment [55]. Early-stage neurons appear as round bodies, in which the growth of actin-rich filopodia and lamellipodia marks the step to the acquisition of cellular polarity [56]. Stabilization by microtubules leads to the development of neurites, which then differentiate into axons and dendrites as the cell acquire their mature neuronal morphology [55]. Proper neurite formation is essential for establishing neuronal morphology such as arborization and synapse formation, which in turn influences connectivity in the brain. The same guidance molecules play an important role in directing axonal growth and influencing synaptic plasticity during development and later in life [57]. As such, variants in axon guidance genes have been implicated in both developmental conditions and neurodegenerative diseases. Cumulative evidence pointed out inappropriate connectivity due to abnormal neuronal density, dendritic arborization and/or cortical layering as one of the causes of ASD [58, 59]. Disturbances of neuritogenesis is also a recurring leitmotif in motor disorders with predominant pyramidal tract dysfunction. Variants in proteins involved in microtubule dynamics (*SPAST*), axonal maintenance (*ATL1*) and transport (*KIF1A*) are among the most common genetic etiologies both of cerebral palsy mimicries [20] and of hereditary spastic paraplegia (HSP) [60]. HSPs are progressive, neurodegenerative disorders with later, often adult, onset in many cases [61]. Spasticity in the lower limbs is the most prominent clinical sign, which can occur isolated or in combination with several other neurological features [61]. Notably, early onset with protracted clinical stability has previously been identified as an endophenotype in a subset of patients in HSP families, resembling the non-progressive course of CP [61].

The selective susceptibility of motoneurons to defect of neuritogenesis is plainly explained by their characteristic morphology: extremely long axons with extensive terminal branching. This pose exceptional challenges for the targeted delivery of presynaptic components from the soma, where they are mostly synthetized, as well as for the removal of defective organelles which must be retrogradely transported. Motor proteins such as kinesins and dyneins, along with several adaptors and scaffolding elements, are in charge of the bidirectional transport of synaptic cargos to ensure precise assembly, maintenance, and remodeling of synapses [62]. At least 23 genes coding for such cargo machinery have been associated with NDDs [62]. A particularly broad phenotypic spectrum is associated with variants in the *KIF1A* (MIM *601255) [63], a kinesin responsible for the anterograde transport of synaptic vesicle precursors along axonal and dendritic microtubules (see also Table 1) [63]. More than 100 disease-associated *KIF1A* variants have been described in the literature [64]. The broad spectrum of clinical symptoms encompasses both neurodevelopmental and neurodegenerative categories such as developmental delay, intellectual/learning disability, autism, epilepsy, microcephaly, spastic CP, HSP, peripheral neuropathy, optic nerve atrophy, and cerebellar atrophy [64–68]. Considering that anterograde transport of presynaptic components is required for both development of the brain and maintenance of axons functionality through life, this variability is not surprising. *KIF1A* variants can be dominantly and recessively inherited, as in HSP families, or appear *de novo* in the most severe phenotypes [64, 69]. Some further genotype-phenotype correlations are known. For instance, patients carrying missense variants at the position 13 (such as R13H and R13C) are at a high risk of ASD [65, 67].

### Synaptic Dysfunction in Focus: the Example of *CACNA1A* Disease Spectrum

Proper neural morphogenesis and branching is instrumental to the development of brain connectivity. The human central nervous system contains ∼10^15^ synapses between ∼10^12^ neurons, building a hyper-wired interconnectome [70]. Intense synaptogenesis occurs during embryonic and early postnatal stages, persists throughout adolescence and up to the third decade [71]. Not surprisingly, disruption of synaptic transmission and plasticity leads to a wide range of NDDs. In this regard, synaptic ion channel dysfunction is particularly associated with epileptic encephalopathies, devastating neurological disorders characterized by early onset of multiple seizure types, psychomotor regression, and a variety of focal neurological signs [72]. A prototype is Dravet syndrome (MIM #607208), in which loss-of-function variants of *SCN1A*, a sodium channel gene expressed almost exclusively in inhibitory interneurons, result in network hyperexcitability [73]. Less straightforward are the molecular and cellular mechanisms underpinning the developmental cognitive and motor impairments associated with early-onset channelopathies [74]. Altered synaptic plasticity during early cortical development likely contributes to the disease phenotype.

**Fig. 1:**
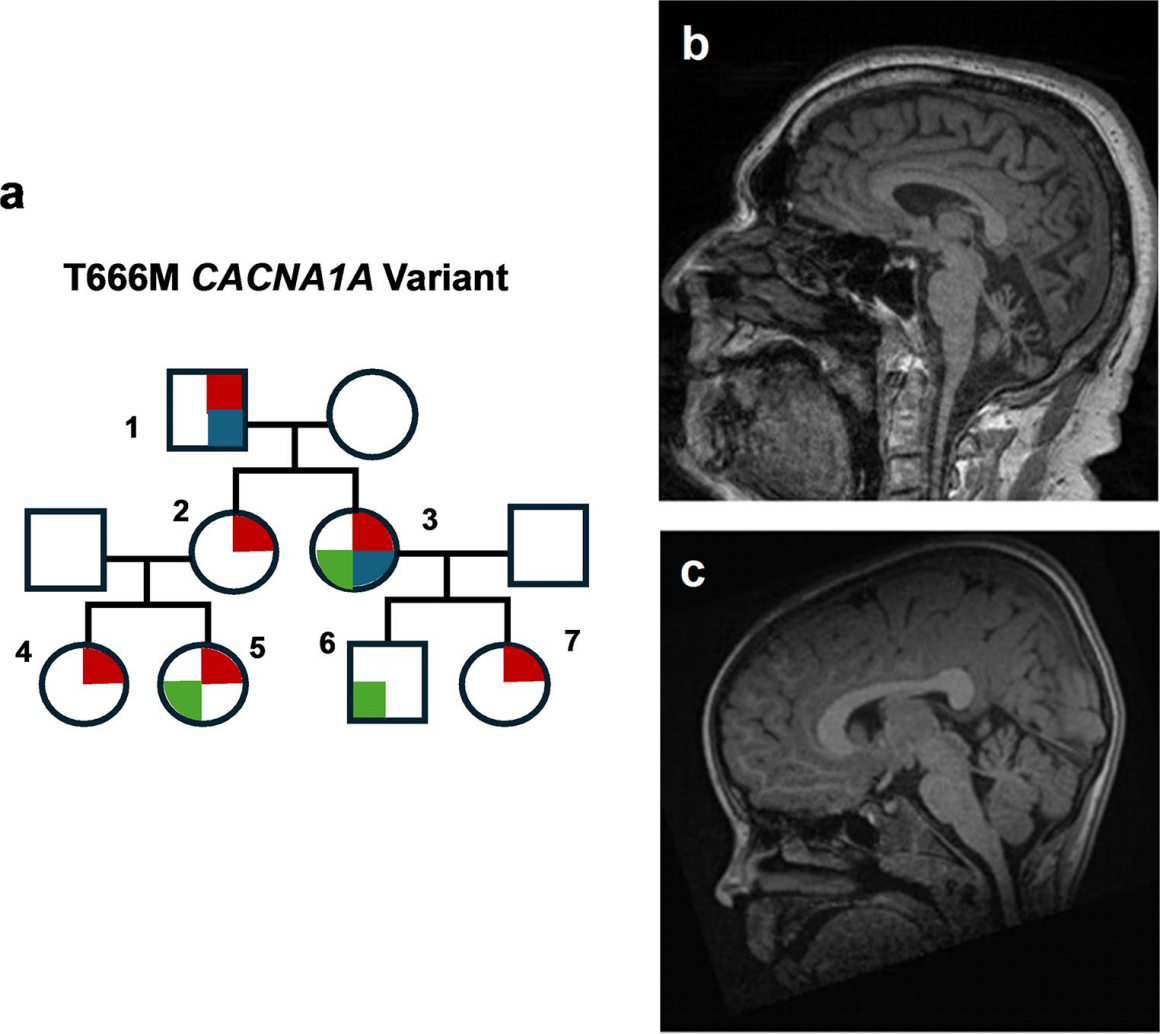
Neurodevelopmental dysfunction and neurodegeneration in *CACNA1A* variants. Panel (**a**) shows a *CACNA1A* pedigree and its clinical spectrum across generations (red upper right quadrant: hemiplegic migraine, blue lower right quadrant: progressive cerebellar ataxia, green lower left quadrant: developmental delay). The index patient (subject 1 in Panel **a**) suffered from hemiplegic migraine since his teens and later developed progressive cerebellar ataxia with clear evidence of neurodegeneration on brain imaging (panel **b**: T1-weighted sagittal plane shows marked cerebellar atrophy, most pronounced in the vermis). The youngest family members (subjects 5 and 6 in panel **a**) were initially referred for developmental delay; patient 5’s brain MRI (panel **c**) was unremarkable

With respect to motor dysfunction, channelopathies are a classic etiology of paroxysmal movement disorders [75] as well as neurodegenerative diseases such as hereditary cerebellar ataxias [76]. In hereditary ataxias, Purkinje neurons in the cerebellum are particularly susceptible to degeneration. Notably, Purkinje cells are autonomous pacemaker neurons that maintain firing at 40 Hz even in the absence of synaptic input [77]. Thus, perturbations in ion channel expression and function have the potential to profoundly affect these neuronal types and cause motor impairment [78]. The *CACNA1A* disease spectrum is paradigmatic for a channelopathy at the interface between neurodevelopmental dysfunction and neurodegeneration (see Fig. 1; Table 1). *CACNA1A* (MIM *601011) is a bicistronic gene which encodes both the pore-forming α1A-subunit of the neuronal P/Q Ca^2+^ channel [79] and the transcription factor α1ACT which drives maturation of the Purkinje cells in the early development [80]. The first association of *CACNA1A* with human disease dates back to 1996 [32, 81]. On the one hand, it contains a CAG repeat motif that can undergo expansion, causing spinocerebellar ataxia type 6, a well-characterized, pure cerebellar disorder with onset in the 5th -6th decade [81]. On the other hand, single nucleotide variants (SNVs) in *CACNA1A* have been associated with a variety of other phenotypes featuring both chronic cerebellar and neuropsychiatric symptoms as well as episodic manifestations, ranging from hemiplegic migraine to epilepsy [31, 82–85]. After the initial association of *CACNA1A* SNVs with human disease in a landmark study [32], a number of reports noticed the recurrence of early onset phenotypes with developmental delay, intellectual disability, ADHD, ADS in the offspring of *CACNA1A* families (see Fig. 1) [83]. With the advent of large-scale exome sequencing-based genetic studies, *de novo* SNVs in *CACNA1A* have been definitely pinpointed as a relevant etiologies in the NDDs spectrum, for developmental and epileptic encephalopathies [41] as well as CP [20]. Complemented by the first large-scale clinical registry [86], an extreme phenotypic variability of “non-polyglutamine” *CACNA1A* disorders is emerging, with genotype-phenotype correlations being difficult to discern so far [86]. Taken together, these cumulative findings suggest an age-dependent phenotype of *CACNA1A* variants, in which the clinical severity is associated with a disease onset early in life and *de novo* occurrence [87, 88].

Early studies in mice highlighted the importance of P/Q channels for the firing activity of Purkinje neurons, providing a pathophysiological correlate for the motor dysfunction seen in human disease. In healthy conditions, autonomous spiking in Purkinje neurons is very precise, with roughly constant duration of interspike intervals. In ataxic mice carrying *CACNA1A* variants, Purkinje neurons show irregular spiking compared to wild-type controls, as evidenced by an increase in the coefficient of variation of the interspike interval between action potentials [78, 89]. In contrast, the pathophysiological basis of NDDs due to pathogenic *CACNA1A* variants remains largely unexplored [90]. Both P/Q calcium currents and the transcription factor α1ACT have established roles in the early cerebellar maturation. Perturbations in the developing cerebellum underpinned by P/Q channel and α1ACT dysfunction may contribute to the onset of neuropsychiatric disorders early in life by altering cerebellar tuning to cognitive cortical networks, consistent with the notion of a “cerebellar cognitive-affective syndrome” [91, 92].

**Table 1:** Genes at the intersection between NDDs and movement disorders

Gene MIM number	Phenotype	Phenotype MIM number	Inheritance
*NKX2-1* *600635	Chorea, hereditary benign	118700	AD
	Choreoathetosis, hypothyroidism, and neonatal respiratory distress	610978	AD
*KMT2B* *606834	Dystonia 28, childhood-onset	617284	AD (*de novo* variants in most patients)
	Intellectual developmental disorder, autosomal dominant 68	619934	AD (*de novo* variants in most patients)
*KIF1A* *601255	NESCAV syndrome	614255	AD (*de novo* variants)
	Neuropathy, hereditary sensory, type IIC	614213	AR
	Spastic paraplegia 30, autosomal dominant	610357	AD
	Spastic paraplegia 30, autosomal recessive	620607	AR
*CACNA1A* *601011	Developmental and epileptic encephalopathy 42	617106	AD (*de novo* variants in most patients)
	Episodic ataxia, type 2	108500	AD
	Migraine, familial hemiplegic, 1	141500	AD
	Migraine, familial hemiplegic, 1, with progressive cerebellar ataxia	141500	AD
	Spinocerebellar ataxia 6	183086	AD

Selected genes implicated both in NDDs and movement disorders are listed along with the associated phenotypes and mode of inheritance. AD: autosomal dominant; AR: autosomal recessive; MIM: Mendelian inheritance in man; NESCAV: NEurodegeneration-Spasticity-Cerebellar Atrophy-cortical visual impairment syndrome

### NDD Genes in Movement Disorders: Insights from Genetic Studies in Dystonia

Among movement disorders, dystonia shares perhaps the greatest genetic overlap with NDDs [9]. Dystonia is highly heterogeneous in terms of phenomenology, comorbidity, and underlying pathogenic mechanisms [93]. In contrast to other movement disorders, neuropathological studies in most monogenic dystonias have not demonstrated any consistent structural brain abnormalities or neurodegeneration [94]. Instead, cumulative evidence supports the concept of dystonia as a network disorder, arising from dysfunctional connectivity involving several brain regions [95]. In recent years, the application of unbiased exome sequencing in large cohorts revealed an even greater heterogeneity in its genetic landscape [21, 96]. Key findings were provided by a landmark study of 764 unselected index patients with variable manifestations ranging from (i) isolated dystonia to (ii) dystonia “combined” with other movement disorders or (iii) “complex” dystonia associated with other non-movement disorder neurological features [21]. Unbiased exome sequencing yielded a genetic diagnosis in 135 of 764 index cases (19%). Notably, the majority of diagnoses (*n* = 94, 69.6%) were related to variants in genes previously associated with NDDs [21]. These included classic genes first characterized in ASD (*MECP2*,* CHD8*,* SHANK3*), intellectual disability and global developmental delay (*AUTS2*, *ZMYND11*, *ZEB2*,* SLC9A6*,* PPP2R5D*,* PAK1*). As expected, the largest contribution of variants in NDD-associated genes was found in cases with “complex dystonia”, who had a variety of developmental disabilities and other associated features. However, even in cases of isolated and combined dystonia, up to one third of the diagnoses were due to variants in NDD genes. Based on this study and its validation [21, 96], a scoring algorithm has been outlined to guide the choice of genetic testing in dystonia. This algorithm lists as positive predictors for the yield of genetic testing: (i) a higher severity of the clinical syndrome of dystonia (generalized versus focal/segmental), (ii) the association with other movement disorders or non-movement disorder neurological features, and (iii) a younger age at onset (< 21 years) [21]. With increasing score, both the yield of exome sequencing and the percentage of diagnoses attributable to NDD-associated genes increase [96].

## Conclusion

Brain disorders have traditionally been classified into early-onset neurodevelopmental and late-onset neurodegenerative disorders [97]. Much of this dogmatic separation is due to the dichotomous approach of clinicians, as pediatricians deal with the neurodevelopmental phase of the disease, whereas the later neurodegenerative phase is managed by adult neurologists [98]. Instead, the shared cellular and molecular processes involved in both neurodevelopmental and neurodegenerative disorders are increasingly recognized [34, 97]. It is also becoming clear that the pathogenesis of some classic neurodegenerative diseases is associated with neurodevelopmental aberrations [99, 100]. Furthermore, an abnormal development may set the stage for selective vulnerability of specific neuronal populations in later-onset degenerative diseases.

Notwithstanding the increasing association between the two clinical constructs at the genetic level, the movement disorder phenotype in several NDDs is often poorly recognized or underreported due to clinical complexity. Identifying a movement disorder in NDDs is important as it has a major impact on quality of life and symptom management [101, 102]. Moreover, the syndromic nature of a clinical presentation with both a movement disorder and neurodevelopmental features represents a hint for a monogenic etiology [10, 103, 104]. Proper characterization of movement disorders early in life may be challenging [102]. Inter-rater agreement for movement disorders in infancy is poor [102]. On the other hand, it may also be difficult to recall subtle abnormalities early in life in a subject who presents for the first time to the neurological clinic in adulthood. In general, lack of awareness and perceived low clinical benefit have limited access of adult cohorts to screening projects for unsolved rare diseases [105]. Another increasingly recognized challenge is furthermore posed by the evolving nature of neurologic manifestations, which often depend on the specific time point in life [106, 107]. Anecdotal reports highlight this issue, showing that delayed diagnostic work-up in adulthood was only triggered by the eventual emergency of a specific movement disorder [10]. This emphasizes the importance of ongoing, regular neurological surveillance, especially during the transition from the child-centered to adult-centered health care [98]. Accurate characterization of the long-term natural history of rare neurogenetic disorders is critical, as it may facilitate the identification of novel molecular targets that are relevant early in the disease course and allow the subsequent development of truly disease-modifying interventions. Just as the clinical evaluation must be regular and thorough, the genetic approach at the time of diagnosis must be comprehensive. Most importantly, the genes investigated in movement disorders should include those associated with NDDs [108].

Elucidating the common biological basis of neurodevelopmental dysfunction and neurodegeneration is a critical step in formulating a translational approach that promotes potential therapeutic strategies. Relevant aspects of neurodevelopmental dysfunction that may also play a role in later disease involve dynamic changes related to gene expression and epigenetics. Modulation of these phenomena [15, 109] may open a window of opportunity for therapeutic interventions before progressive degeneration and structural brain damage manifest.

## Key References

• • Gonzalez-Mantilla PJ, Hu Y, Myers SM, et al. (2023) Diagnostic Yield of Exome Sequencing in Cerebral Palsy and Implications for Genetic Testing Guidelines: A Systematic Review and Meta-analysis. JAMA Pediatr 177:472. 10.1001/JAMAPEDIATRICS.2023.0008.

Metanalysis of exome sequencing studies in cerebral palsy, showing (i) an average diagnostic yield of 31.5% and (ii) recurrent etiologies among genes traditionally associated with movement disorders.

• • Zech M, Jech R, Boesch S, et al. (2020) Monogenic variants in dystonia: an exome-wide sequencing study. Lancet Neurol 19:908–918. 10.1016/S1474-4422(20)30312-4.

Large whole exome sequencing study in dystonia showing that two thirds of diagnosis are attributable to neurodevelopmental genes.

• Hickman RA, O’Shea SA, Mehler MF, Chung WK (2022) Neurogenetic disorders across the lifespan: from aberrant development to degeneration. Nat Rev Neurol 18:117. 10.1038/S41582-021-00595-5.

Review on common molecular pathways across neurodevelopmental and neurodegenerative disorders.

• Cunha P, Petit E, Coutelier M, et al. (2023) Extreme phenotypic heterogeneity in non-expansion spinocerebellar ataxias. Am J Hum Genet 110:1098–1109. 10.1016/J.AJHG.2023.05.009.

Large clinicogenetic study in hereditary ataxias highlighting an extreme phenotypic variation ranging from early onset severe developmental phenotypes to slow progressive degenerative cerebellar dysfunction.

## Data Availability

No datasets were generated or analysed during the current study.
